# Development of four-bar polycentric knee joint with stance-phase knee flexion

**DOI:** 10.1038/s41598-023-49879-4

**Published:** 2023-12-20

**Authors:** Santiphap Phoengsongkhro, Pairat Tangpornprasert, Pattarapol Yotnuengnit, Manunchaya Samala, Chanyaphan Virulsri

**Affiliations:** 1 Center of Excellence for Prosthetic and Orthopedic Implant, Chulalongkorn University, Bangkok, 10330 Thailand; 2 Department of Mechanical Engineering, Faculty of Engineering, Chulalongkorn University, Bangkok, 10330 Thailand; 3 Biomedical Engineering Research Center, Faculty of Engineering, Chulalongkorn University, Bangkok, 10330 Thailand; 4 Department of Rehabilitation Medicine, Faculty of Medicine, Chulalongkorn University, Bangkok, 10330 Thailand; 5 Sirindhorn School of Prosthetics and Orthotics, Faculty of Medicine Siriraj Hospital, Mahidol University, Bangkok, 10700 Thailand

**Keywords:** Health care, Engineering

## Abstract

A conventional 4-bar polycentric knee and solid ankle cushion heel foot (SACH foot) have been commonly used in developing countries. However, they cannot perform stance-phase knee flexion, which makes a person with an amputation walk unnaturally and with less stability. This research proposes a novel design of a 4-bar polycentric knee with stance-phase knee flexion ability (4BSF), which can perform both stance and swing-phase knee flexion, like able-bodied gait. In the proposed conceptual design, the instantaneous center of rotation (ICR) path is repositioned during the stance phase. The ICR was placed in front of the ground reaction force (GRF) to initiate knee flexion during the loading response. The prototype was validated by a single-subject pilot study at the Gait analysis laboratory. The results showed that a person with an amputation walks with stance-phase knee flexion using the proposed 4BSF. The maximum knee flexion angle is more than 10° during the stance phase. Furthermore, when the 4BSF was used with a SACH foot, the amount of time to achieve the foot flat was shorter, and the foot flat duration time was twice as long as the conventional 4-bar polycentric knee.

## Introduction

Nowadays, there are 40 million people with amputations in low- and middle-income countries. However, only 17.7% of people with amputations can access lower limb prostheses^1,2^. Moreover, an additional cost is incurred to reach a living standard to be equivalent to the able-bodied^3^. When people with amputations decide to purchase a prosthetic knee, there are many crucial factors for consideration, i.e., cost, durability, maintenance, and stance stability^4^. Due to the cost constraints, purely mechanical prosthetic knees, such as single-axis knee joints and 4-bar polycentric knees, are commonly used in developing countries^5^.

Current prosthetic knee technology has a versatile design. The least complicated prosthetic knee is a single-axis knee joint. A hinge joint mechanism is used, resulting in easy knee joint flexing. People with amputations who use single-axis knee joint must exert their hip extension moment to maintain knee stability^6,7^. Subsequently, single-axis knee joints were improved by adding a lock control axis called the 2-axis knee joint. This improved stance stability by allowing the knee to flex during the swing phase only when the ground reaction force passes between its two pivot points^8–10^. The 3-axis knee joint was later developed from the 2-axis knee joint by adding a pivot for flexion during the stance phase. This helped people with limb loss walk more naturally when their knees flexed during the stance phase^11,12^. However, knee joints with an added a lock control axis are difficult to flex during the swing phase^10^.

Another type of prosthetic knee is the polycentric knee. A conventional 4-bar mechanism was adopted in this type of prosthetic knee. It consists of 4 bars assembled in a closed loop. At any instant in time, a 4-bar polycentric knee is rotating around a particular point, called the instantaneous center of rotation (ICR). It is an advantage that the 4-bar mechanism can be designed to better position the ICR point in the stability zone. Therefore, the polycentric knee is more stable than the single-axis knee joint^13^. Additionally, during knee flexion in the swing phase, the 4-bar polycentric knee has a more significant toe clearance gap than the single-axis knee joint due to the change in geometry of the 4-bar mechanism. Using the polycentric knee reduces the chance of people with amputations tripping between their feet and the ground^14–17^. A 5-bar polycentric knee with 2 DOF was invented for stance-phase knee flexion. It is more stable due to the change in ICR position to higher and more posterior during the stance phase^18^. Additionally, a 6-bar polycentric knee with 1 DOF was developed to mimic the ICR path of the human knee joint and to allow knee flexion during the stance phase^19,20^.

One crucial gain that low-end knee joints cannot do is stance-phase knee flexion, which allows people with limb loss to walk like the able-bodied. Stance-phase knee flexion decreases impact forces and absorbs energy while walking^21^. The following effect is a reduction in vertical hip movement and mobility, which reduces oxygen consumption^22,23^. Normally, stance-phase knee flexion can be performed on 5-bar and 6-bar knee joints. However, people with amputations in developing countries cannot access these knee joint systems because of financial limitations, a substantial number of parts, and the intricacy of the mechanisms. Increasing the number of parts and joints elevates both mass production costs and maintenance expenses. In addition, most people with limb loss use a solid ankle cushion heel (SACH) foot^24^. Due to the lack of ankles, the foot flat occurs very slowly with less contact area between the foot and the ground resulting in less stability^25–27^. The stance phase knee flexion may increase foot stability in the missing ankle of the SACH foot during the stance phase.

For all the current prosthetic knees mentioned above, the 4-bar knee mechanism is the most suitable for developing countries due to its acceptable stability, use of less hip moment control when compared with single-axis knee joints, high toe clearance, and ease of maintenance. However, the limitation of the conventional 4-bar polycentric knee joint is that the mechanism is still unable to perform stance-phase knee flexion^18^. This research proposes a novel design of a 4-bar polycentric knee with focusing on stance-phase knee flexion ability (4BSF). The functionality of the knee joint mechanism will be validated by a single-subject pilot study. The knee flexion and foot-to-ground angles during walking with the 4BSF will be compared with an able-bodied gait and conventional 4-bar mechanism. All performance tests were carried out with SACH foot, commonly used in developing countries. The new 4-bar polycentric knee design has the potential to increase the stability limits of conventional 4-bar mechanisms during the stance-phase, providing greater versatility and potentially improving stability while walking.

## Design specifications and conceptual design

To design a novel 4-bar polycentric knee with high stability and focus on stance-phase knee flexion function, the design of the 4BSF has important requirements, as follows.

### The path of ICR only moves in the stability zone

The stability zone is the voluntary control zone of the person with an amputation which defines the region of ICR where hip moment can effectively control the stability^13^. The hip moment, which the GRF determines at the heel strike and toe-off, impacts each individual’s stability zone. Therefore, the ICR path of the 4-bar polycentric knee should be located in the stability zone to minimize the chance of falling. The GRF data to define the stability zone to design of 4BSF was taken from Gabriele Bovi et al*.*^28^.

### Stance and swing-phase knee flexion angle

The maximum stance-phase knee flexion angle of a typical able-bodied gait is generally between 10° and 20°, and the swing phase is 60°–70°^23^.

The maximum stance-phase flexion of some commercial prosthetic knees is between 8° and 12°^18^. In this research, the maximum stance and swing-phase knee flexion angle of 4BSF was specified as more than 10° and 60°, respectively.

### Size and weight of the prosthetic knee

The compact size and weight of the prosthetic knee are significant factors affecting walking^29^. The adult polycentric knee from global manufacturers ranges from 510 to 1230 g^30^. It weighs only 33% of an intact leg^29^.

Typically, the length from the femoral condyles to the medial malleolus of the actual leg is approximately 410 mm^31^. The length connects the moment of inertia and the shape of an object. An increase in length also increases the moment of inertia as well^29^. Reducing the masses and moments of inertia of all segments of the prosthetic leg has a significant impact on hip power, with a maximum reduction of up to 26% in peak hip power during stance and up to 74% in average hip power during swing^29^. The average length of an adult prosthetic knee in stance flexion ranges from 188 to 250 mm^30^. The prosthetic knee length is smaller when compared to an actual leg^31^.

Therefore, the weight of the designed prosthetic knee must not exceed 1230 g to guarantee that people with impaired hip moments can use the 4BSF. Additionally, for people with all-leg lengths to use the device, the length of the designed prosthetic knee is taken from the minimum average length of an adult prosthetic knee (≤ 188 mm).

### Conceptual design of knee mechanism

**Figure 1:**
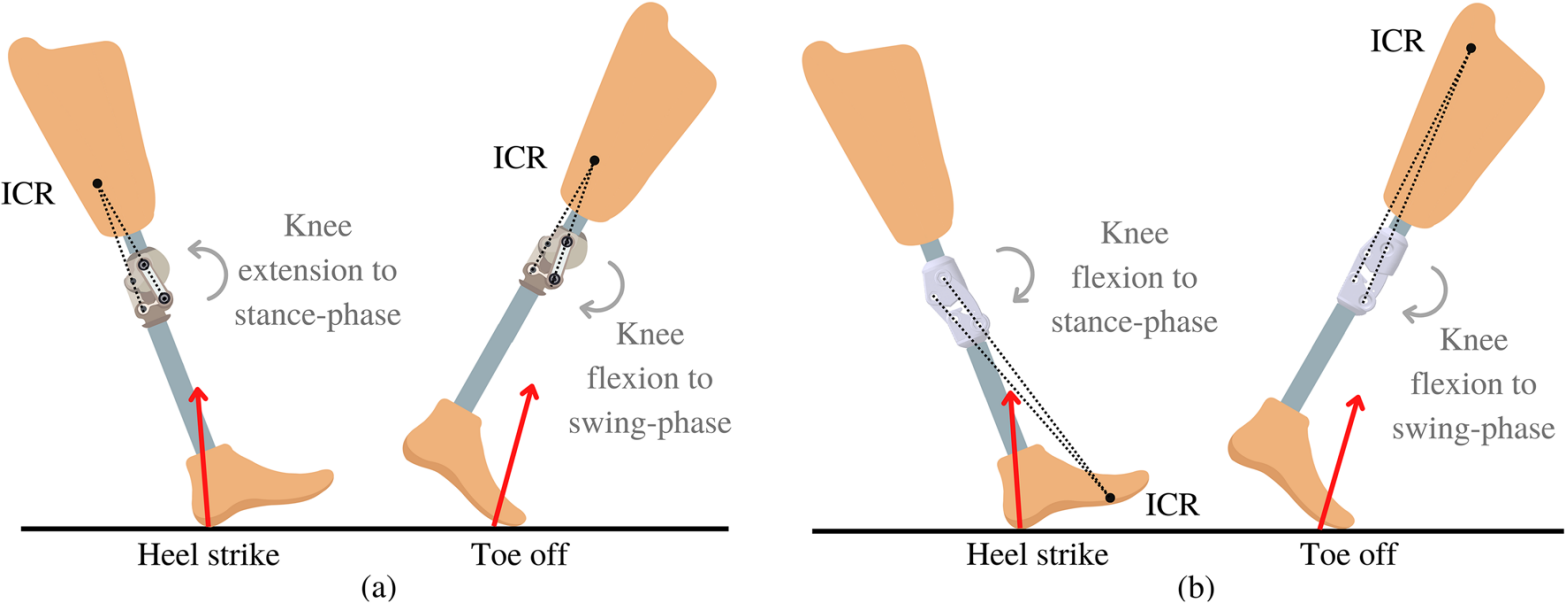
(**a**) Motion of the conventional 4-bar polycentric knee, (**b**) motion of the 4BSF.

The able-bodied have knee flexion during both the stance and swing phases in the same flexion direction. However, in a conventional 4-bar polycentric knee, the GRF during heel strike generates a moment in extension direction, causing the knee mechanism to be an extension which is the opposite direction of the able-bodied. The conventional 4-bar polycentric knee mechanism rotates in flexion during the swing phase as same as the able-bodied, as shown in Fig. 1a.

In contrast, this research proposes a new design that repositions the ICR during the stance phase. The knee mechanism rotated flexion direction to initiate knee flexion during the stance phase after the ICR was positioned in front of the GRF. As a result, the 4BSF flexed the same way as in the swing phase, resembling the able-bodied gait cycle, as shown in Fig. 1b.

## Prosthetic knee design

### Design path of ICR and synthesis

To design a knee mechanism that achieves the design specification. The initial ICR, the stance-phase ICR, and the swing-phase ICR were used to design the ICR path. The complete path of ICR was generated to pass these three points. The conditions used to determine the ICR in each stage are as follows:

#### Initial ICR

**Figure 2:**
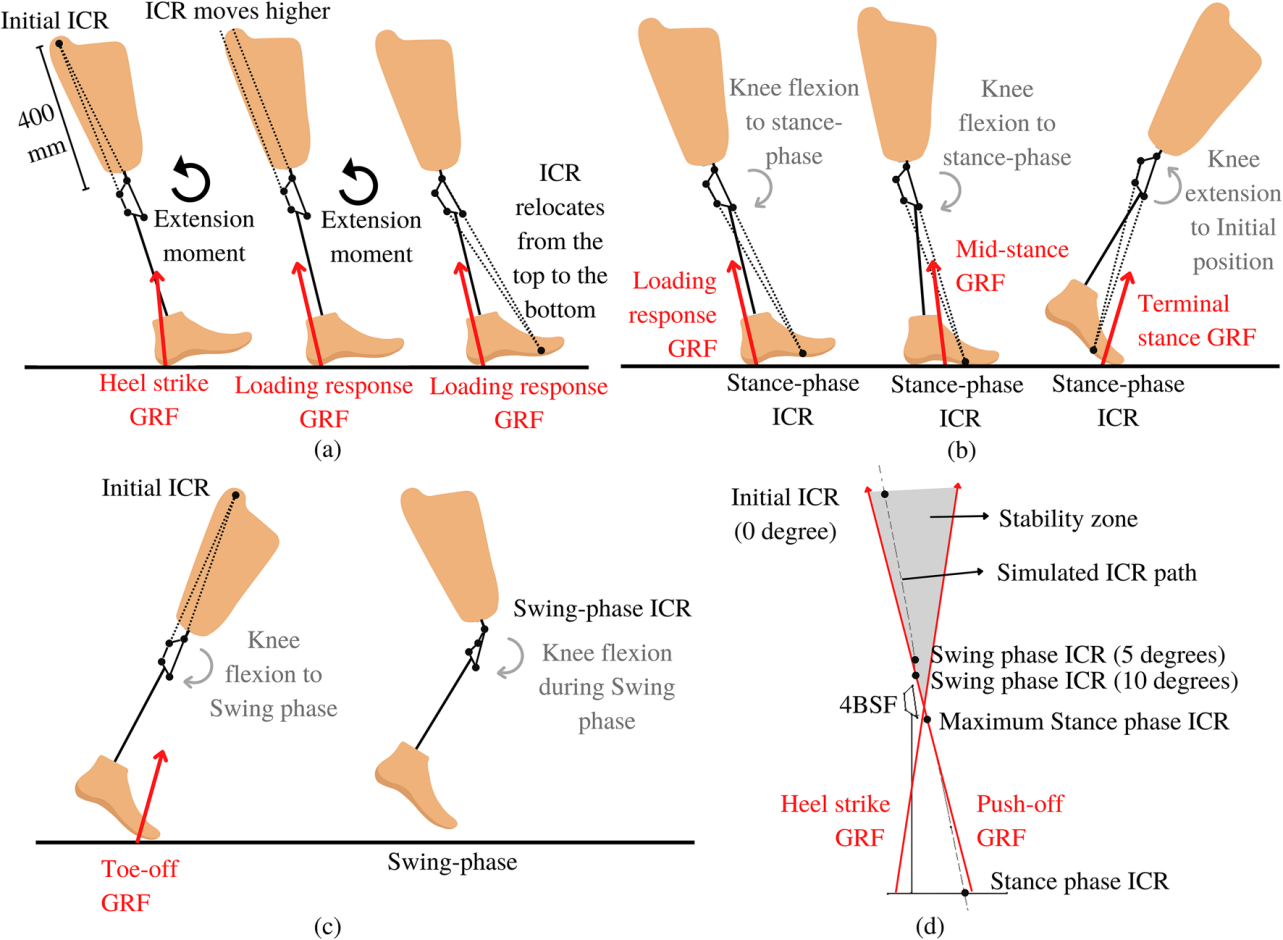
(**a**) Motion of ICR from the initial position to the stance-phase knee flexion, (**b**) stance-phase ICR, (**c**) swing phase, (**d**) swing phase ICR (0°, 5°, and 10°), Simulated ICR path, Stance phase ICR, and Mechanism diagram^13^.

The stability zone is typically where the first ICR is found. This research placed the position of the initial ICR higher than the knee at 400 mm. This position makes the knee achieve high stability but not hyper-stability^14^. As shown in Fig. 2a, linkages of the mechanism were designed to turn parallel quickly. Then, the ICR rapidly relocated to the front of the GRF and achieved stance-phase knee flexion.

#### Stance-phase ICR

Throughout the stance-phase, the ICR of the conventional 4-bar was located in the exact location of the beginning position. In contrast, the ICR of the 4BSF was designed to move during the stance-phase. In the changing phase from heel strike to loading response (the GRF rapidly increases in magnitude, with an upwards and backwards direction^23^), the ICR was moved from the top to the bottom and on the front side of the GRF. Moreover, stance-phase ICR was located in front of GRF during mid-stance. The polycentric knee continues to move to the maximum stance phase knee flexion until it comes into contact with the stopper. The ICR at this position corresponds to the maximum stance phase ICR, as shown in Fig. 2d. Then, the GRF moves to the front of the stance-phase ICR during the terminal stance, as shown in Fig. 2b.

#### Swing-phase ICR

The swing-phase ICR in the 4BSF was located in the stability zone, which is also in the same position as the initial ICR. Therefore, the GRF is behind this ICR during toe-off, causing the knee mechanism to rotate flexion direction toward the swing-phase knee flexion, similar to the conventional 4-bar mechanism.

Additionally, this design considered safety throughout the terminal swing. If there is an unlikely circumstance where the knee mechanism is not fully extended within 10°, the person with an amputation can still control the knee joint mechanism to prevent collapse. As shown in Fig. 2d, the path of ICR during the first 10° of knee flexion was designed to be in the stability zone.

#### Synthesis of the 4BSF mechanism

To synthesize the mechanism for a 4BSF with stance-phase knee flexion, the design specifications can be summarized as follows:

- The ICR path must pass through three ICR points (Initial ICR, Swing phase ICR at 10°, and Stance phase ICR), as shown in Fig. 2d.
- Maximum stance-phase knee flexion angle > 10°
- Maximum swing-phase knee flexion angle > 60°

The 4BSF was synthesized with GIM software version 2022 to achieve the above requirements^32^. The result of the simulated ICR path is shown in Fig. 2d.

### Stability and movement of the 4BSF mechanism in the gait cycle

According to Fig. 2a–c, Stability during the gait cycle of 4BSF is as follows.

**Figure 3:**
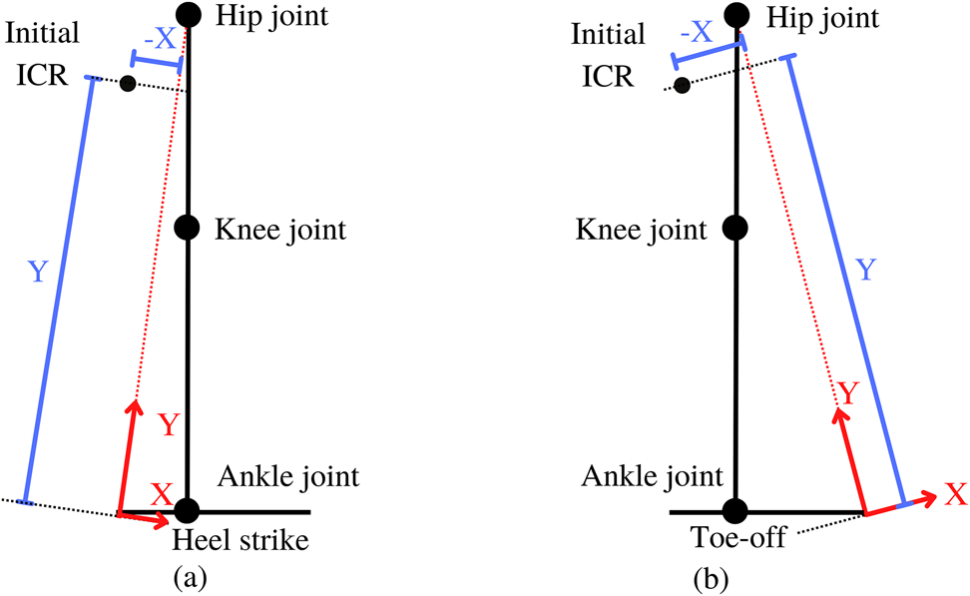
Stability of polycentric knee (**a**) heel contact X/Y ratio, (**b**) push off X/Y ratio^14^.

- Heel strike (Fig. 2a): the knee mechanism is designed to have an initial ICR located at a high position and behind the GRF. Anand et al*. *^14^ demonstrated that when the ICR is located on the Y axis, the polycentric knee will remain stable without requiring the amputee to exert effort with their hip. If the ICR is located in front of the Y axis (x/y ratio = positive), the GRF will create a flexion moment on the knee, causing it to flex. Conversely, if the ICR is located behind the Y axis (x/y ratio = negative), the GRF will generate an extension moment on the knee, causing it to extend, as shown in Fig. 3a.
- Loading response (Fig. 2a): the ICR shift to the front of the GRF, causing the mechanism to rotate to the knee flexion direction until it contacts the stopper. Hence, the mechanism is very stable and does not collapse.
- Mid-stance & Terminal stance (Fig. 2b): GRF shift to the front of ICR, which changes the moment of GRF about ICR to extend the knee mechanism. Furthermore, the knee mechanism rotates into its initial position due to the moment induced by the spring.
- Toe-off (Fig. 2c): ICR moves back to its initial position in the stability zone. The Push-off x/y ratio is also close to zero, making the mechanism easier to swing^14^, as shown in Fig. 3b.
- Swing (Fig. 2c): the mechanism rotates flexion direction about ICR to flex the knee in the swing phase. Toe clearance is increased during the mid-swing in the same way as the conventional 4-bar mechanism^14^. In the terminal swing, the mechanism is designed to have an ICR in the stability zone. This allows the user to control the stability. The mechanism does not collapse in a situation when it has still not reached full extension during a heel strike.

### Detailed design

**Figure 4:**
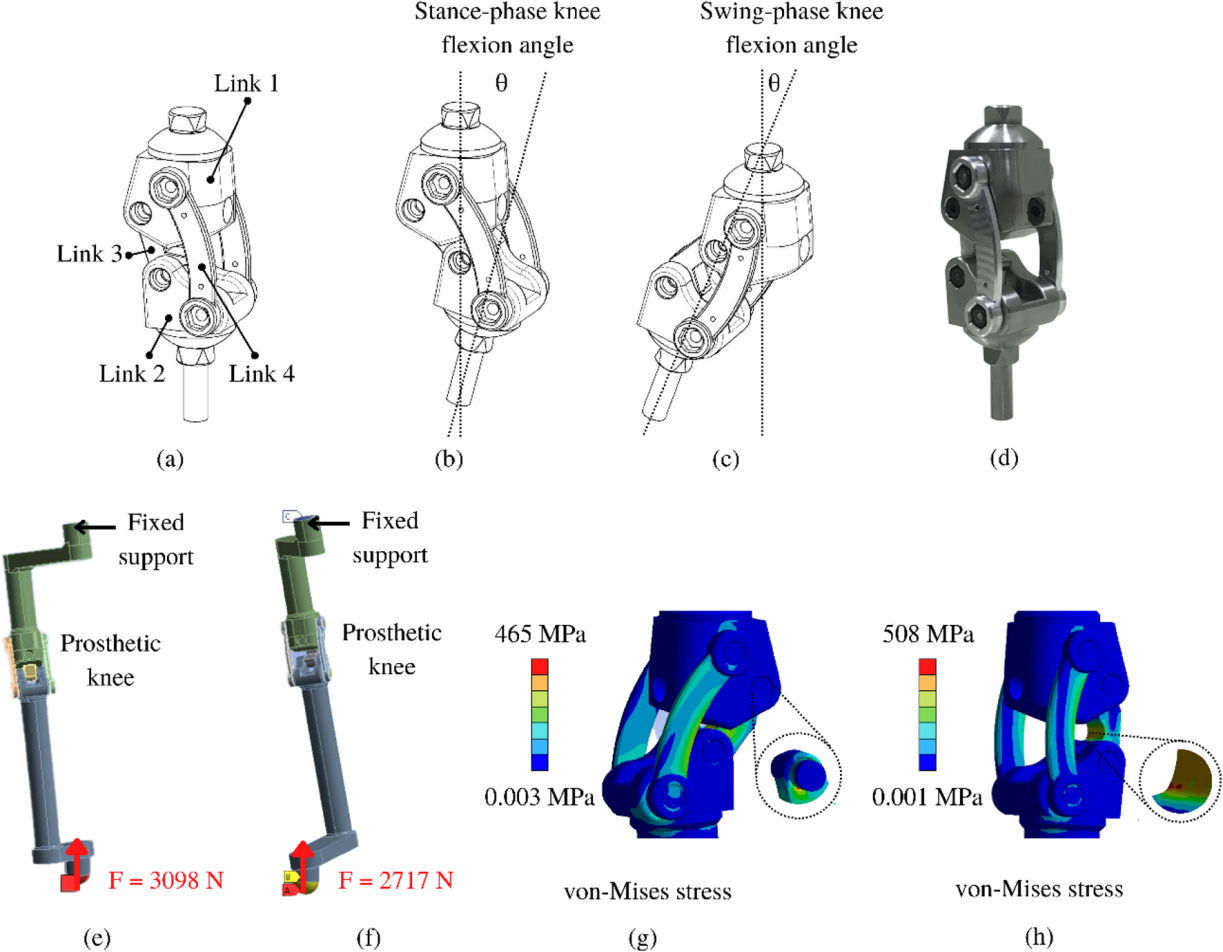
(**a**) The structure of 4BSF, (**b**) configuration of 4BSF during stance phase knee flexion, (**c**) configuration of 4BSF during swing phase knee flexion, (**d**) prototype of 4BSF, (**e**) Boundary conditions of testing loading level P4 and test loading condition I, (**f**) boundary conditions of testing loading level P4 and test loading condition II (**g**) von-Mises stress result of ultimate static test force under loading condition I, (**h**) von-Mises stress result of ultimate static test force under loading condition II.

The structure of the prosthetic knee is composed of four linkages connected with four pivots into a closed loop, as shown in Fig. 4a. Link 1 is attached to the socket. Link 2 is attached to the pylon. Link 3 and 4 hold the entire structure together with the following details.

- Link 1 has a pyramid adapter used to connect with the socket and set up the alignment for each person with an amputation. Therefore, stainless steel was selected, suitable for wear resistance while installing the alignment using screws. Additionally, the stance-phase spring was intended to fit inside the space provided by this link.
- Link 2 also has a pyramid adapter. Thus, stainless steel was used. Moreover, a longitudinal hole was drilled to locate the swing-phase spring.
- Link 3 is essential for prosthetic knee design because it assists the mechanism of returning to full extension (the initial position). During the terminal-stance, the mechanism returns to full extension by the stance-phase spring. During the swing-phase, the mechanism returns to full extension by the swing-phase spring. Both springs can adjust the pre-tension and change the spring stiffness as required.
- Link 4 is made from Aluminum 7175 material because it is lightweight, economic, high strength, and resistant to fatigue^33^.
- Bronze bushings were installed between all joints of the 4BSF.

The configurations of 4BSF mechanism during stance and swing phase knee flexion are shown in Fig. 4b,c respectively. The strength of the 4BSF was tested using the Finite Element (FE) solver ANSYS Workbench 2020 R1 with static structural analysis. The boundary conditions and for test loading condition I (Heel strike) and II (Toe off) with test loading level P4 (weight ≤ 80 kg) followed the ISO 10328 standard^34^, as shown in Fig. 4e,f. The prototype was tested for both ultimate static test force and cyclic test. These tests were conducted at the maximum knee flexion angle during stance phases under loading condition I and 0° flexion under loading condition II, using a Tetrahedron mesh with a mesh size of 1 mm, 1,421,766 elements, and 1,366,539 nodes. The type of contact modeling had frictionless of all contact. The spring and bushing were not modeled as they are not the main structural components. The finite element mesh was refined until the results reached convergence stability. The prototype passed the ultimate static force test with the maximum von Mises stress under loading conditions I and II measuring 465 MPa and 508 MPa as shown in Fig. 4g,h respectively. These values are below the Tensile Yield Strength of the Aluminum 7175 used in the prototype, which is 524 MPa^33,35^. Moreover, the prototype passed the cyclic test, following Soderberg's failure criterion of nf (1.86) > 1, which ensures that the knee joint will not break due to fatigue.

## Single-subject pilot study

This research focuses on the design and development of 4BSF. The aim of testing is not a clinical trial, but it is to prove the conceptual design. Therefore, it is necessary to collect gait data from people with amputations, and the IRB approved one test in person with an amputation. In the future, the 4BSF will be tested for a larger number of subjects after the results are consistent with the study objectives.

The prototype of the prosthetic knee as shown in Fig. 4d was tested for the gait parameters for a person with an above-knee amputation. The ICR and GRF data of 4BSF were collected to prove conceptual design by comparing the ICR position and GRF vector of the design and experimental result. In addition, the comparison of performance between the 4BSF and conventional 4-bar polycentric knee was also evaluated by natural walking and stability. In this research, natural walking was defined as the stance-phase knee flexion angle of able-bodied gait, with a maximum knee flexion of 10°–20°^23^. Stability was also defined in terms of the foot-to-ground angle, which has the potential to generate a stable base of support during the early stance^25–27^. A foot-to-ground angle that is closer to zero results in greater stability. Stance-phase knee flexion angle and the foot-to-ground curve were compared among the conventional 4-bar polycentric knee (Ottobock 3R20), 4BSF, and an able-bodied gait^31^. The walking performance tests were conducted in the Gait and motion analysis laboratory.

This research received an SIRB Protocol no. 834/2564 (IRB4) or certificate of approval COA no. Si 108/2022 for testing with an unilateral transfemoral person with an amputation from Siriraj Institutional Review Board, Mahidol University, Thailand, and the participant accepted and signed the informed consent before enrollment. All methods were carried out in accordance with international guidelines for human research protection such as the Declaration of Helsinki, the Belmont Report, CIOMS Guidelines and the International Conference on Harmonization in Good Clinical Practice (ICH-GCP).

**Figure 5:**
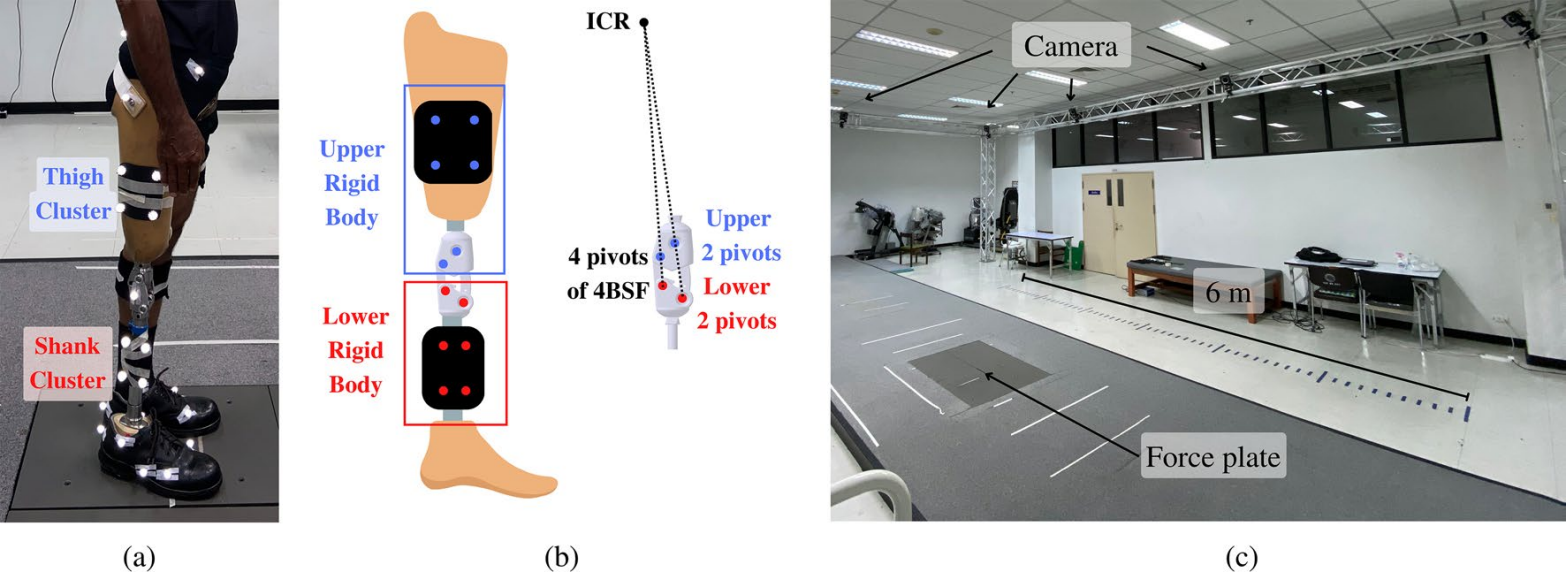
Experiment setup (**a**) reflective markers during walking, (**b**) reflective markers during standing, (**c**) Testing area.

The participant was a 63-year old male who weighted 58 kg with height of 165 cm. He classified as K-level 3 and had used a conventional 4-bar polycentric knee for 10 years. The prosthetic components that he used were a conventional 4-bar polycentric knee, a SACH foot, and a belt socket. A certified prosthetist installed the alignment of the prosthetic leg. Moreover, the participant was trained to use both prosthetic knees for one day prior to the test. The walking motion of the participant was captured by ten motion capture cameras (Optitrack high-speed infrared camera set, Prime 17W model) placed around the testing zone along with a force plate (Bertec, model FP4060-07-1000, four pieces) in the middle of the testing area, as shown in Fig. 5c. The sampling rate of the camera and the force plate were adjusted to 100 Hz for data synchronization. The software for collecting motion data was Motive version 2.0.2, and the software for analyzing movement is VIsual3D version 6. Additionally, reflection markers were attached to the lower body.

To measure stance-phase knee flexion angle and foot to ground angle, the six degrees of freedom model markers were attached to the lower limb, as shown in Fig. 5a^36,37^. The reflective markers were attached to the posterior superior iliac spine, anterior superior iliac spine, greater trochanter, medial epicondyle, lateral epicondyle, lateral malleolus, medial malleolus, heel, first metatarsal, the head of the fifth metatarsal, and the base of the fifth metatarsal. Cluster reflective markers were attached to the thigh and shank. The position of ICR can be determined from the four pivots of 4BSF. To measure the position of ICR, two reflective markers were attached to the two upper pivots of 4BSF, which is the same upper rigid body with a thigh cluster. Two reflective markers were attached to the two lower pivots of 4BSF, which is the same lower rigid body with a shank cluster, as shown in Fig. 5b. After that, the position of all reflective markers was recorded while the participant was standing. Therefore, the relative positions of the cluster and 4BSF markers could be calculated even if four markers on the 4BSF were removed. To prevent interference during walking, the four markers on the 4BSF were removed before walking. After the walking test was complete, the calculated ICR position data was compared to the GRF that occurred during walking to analyze the relation between ICR and GRF.

The walking performance of the 4BSF, the conventional 4-bar polycentric knee, and the able-bodied gait were compared. The walking data of 4BSF and conventional 4-bar polycentric knee were collected from Gait and motion analysis laboratory. The walking data of a typical able-bodied gait with a walking speed was 0.8 m/s were taken from Winter et al*.*^31^.

The conventional four-bar prosthetic knee was installed for the prosthetic knee performance test. After walking for 45 min to get used to the device, the participant walked through the testing area for 6 m at a self-selected speed three times. After that, the participant rested to reduce fatigue. The process was then repeated with the 4BSF, which was tested with three different settings in the stance-phase knee flexion angle by changing the stiffness of the stance-phase spring. High, medium, and low stiffness are 25.7 N/mm, 13.7 N/mm, and 5.48 N/mm, respectively. The swing-phase spring stiffness of the 4BSF is the same spring as the conventional 4-bar knee (Ottobock 3R20). Finally, gait analysis was performed with the data from conventional 4-bar, 4BSF, and the able-bodied gait, which were compared in the next section.

## Results and discussion

The conceptual design of 4BSF was proven by investigating the ICR and GRF movements during the stance-phase. The performances of 4BSF and Conventional 4-bar polycentric knee were evaluated by stance-phase knee flexion angle and foot to ground angle.

### The ICR position and GRF vector during the stance-phase

**Figure 6:**
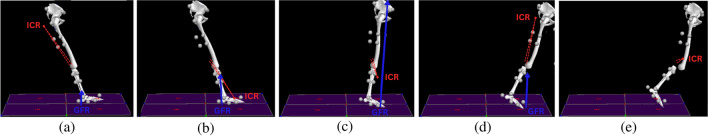
The ICR position and GRF vector of experimental results (**a**) heel strike, (**b**) loading response, (**c**) mid-stance & terminal stance, (**d**) toe-off, (**e**) swing.

From the experimental result, the walking simulation from VIsual3D is shown in Fig. 6. the ICR of 4BSF was in the initial position during the heel strike (Fig. 6a) and in the same position as the conceptual design in Fig. 2a. After that, the ICR moved to the bottom of the prosthetic knee during the loading response (Fig. 6b), like in Fig. 2a, which knee mechanism rotated to stance phase knee flexion because of the flexion moment. Then, GRF shifted to the front of the ICR during mid-stance and terminal stance (Fig. 6c) to extend the knee mechanism in the same result as Fig. 2b. Later, ICR moved back to its initial position during toe-off (Fig. 6d) in order to prepare for the swing phase, the same as in Fig. 2c. Finally, the 4BSF mechanism rotated to swing-phase knee flexion (Fig. 6e), similar to Fig. 2c. The results shown that the position of the ICR and GRF vector of conceptual design and experimental results are consistent.

### Knee flexion angle

**Figure 7:**
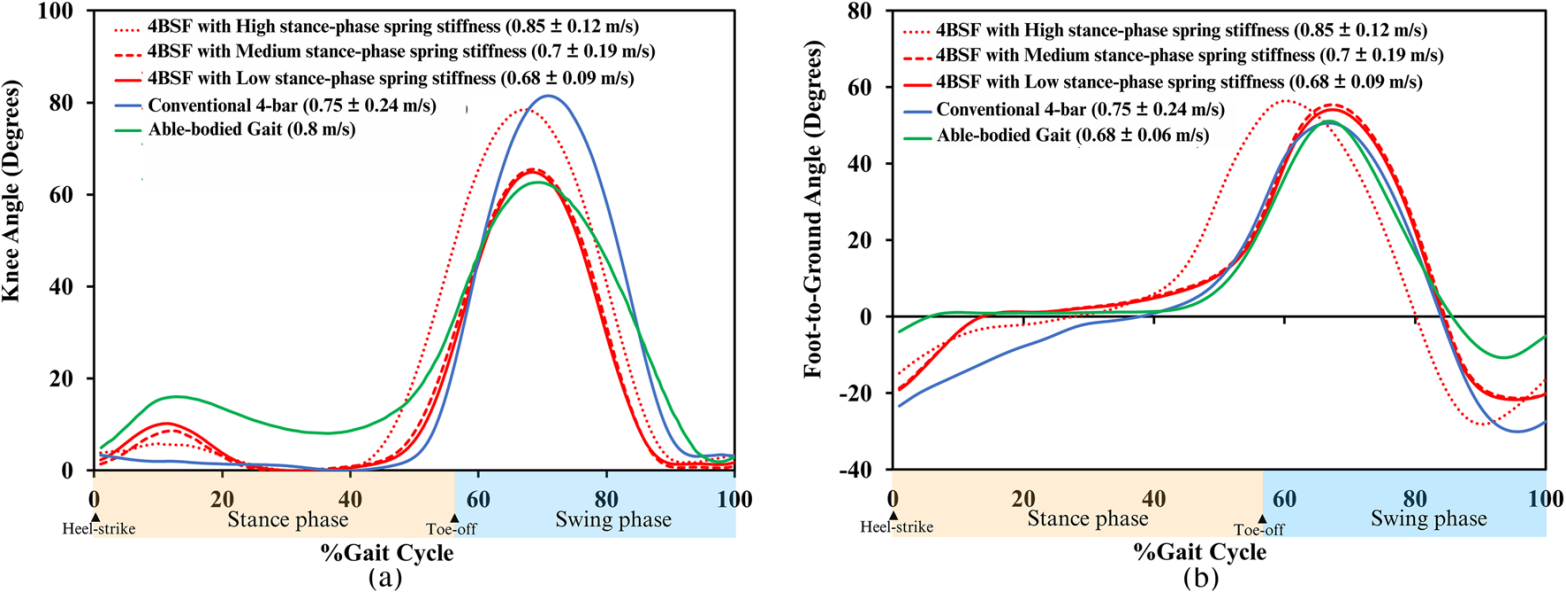
(**a**) Mean prosthetic knee angle for walking trials as a percentage of gait cycles^31^, (**b**) Mean prosthetic foot-to-ground angle for walking trials as a percentage of the gait cycle.

The knee angle data is shown in Fig. 7a. Each data was captured three times, and all three trials were processed. The averaged data with the corresponding standard deviation for the important phases of gait is presented in Table 1. The conventional 4-bar prosthetic knee could not perform a knee flexion throughout the stance phase. On the other hand, the knee angle of the 4BSF started from zero degrees to ensure the person with an amputation’s stability and confidence. The maximum knee flexion angle of the high, medium, and low stance-phase spring stiffness was 5.9°, 8.6°, and 10.2°, respectively. Then, the knee angle returned to 0° at the terminal stance to be ready for the swing phase. When the front and rear bars of the 4BSF mechanism are parallel, the ICR position is located very high and behind the GRF, resulting in increased stability of the mechanism. The period of the front and rear bar going parallel is very short. The participant did not report conscious awareness of this transition. The participant’s comments indicated that walking with the 4BSF felt safer as they did not feel pushed backwards.

**Table 1:** Knee angle in different conditions with standard deviation.

	Knee angle at heel strike (°)	Maximum stance phase knee flexion (°)	Knee angle at full extension (°)	Maximum swing phase knee flexion (°)
4BSF with high stance-phase spring stiffness	3.9 ± 2.1	5.9 ± 0.8	0 ± 0.3	78.5 ± 5.1
4BSF with medium stance-phase spring stiffness	1.4 ± 1.7	8.6 ± 2.9	0 ± 0.4	65.4 ± 4.9
4BSF with low stance-phase spring stiffness	2.3 ± 2.4	10.2 ± 3.0	0 ± 0.3	64.9 ± 7.3
Conventional 4-bar	3.3 ± 0.8	3.3 ± 0.8	0 ± 0.4	81.4 ± 6.2
Able-bodied gait^31^	5	16.1	8.1	62.6

According to Fig. 7a, the stance-phase spring stiffness of 4BSF can be adjusted to the maximum knee flexion angle during stance phases between low, medium, and high settings. However, the magnitude of the maximum stance-phase knee flexion angle depends on the stance-phase spring stiffness. When using a high stance-phase spring stiffness, the resistance to knee flexion increases, resulting in a lower magnitude of maximum stance-phase knee flexion angle. On the other hand, using a low stance-phase spring stiffness results in lower resistance to knee flexion and a higher magnitude of maximum stance-phase knee flexion angle.

**Figure 8:**
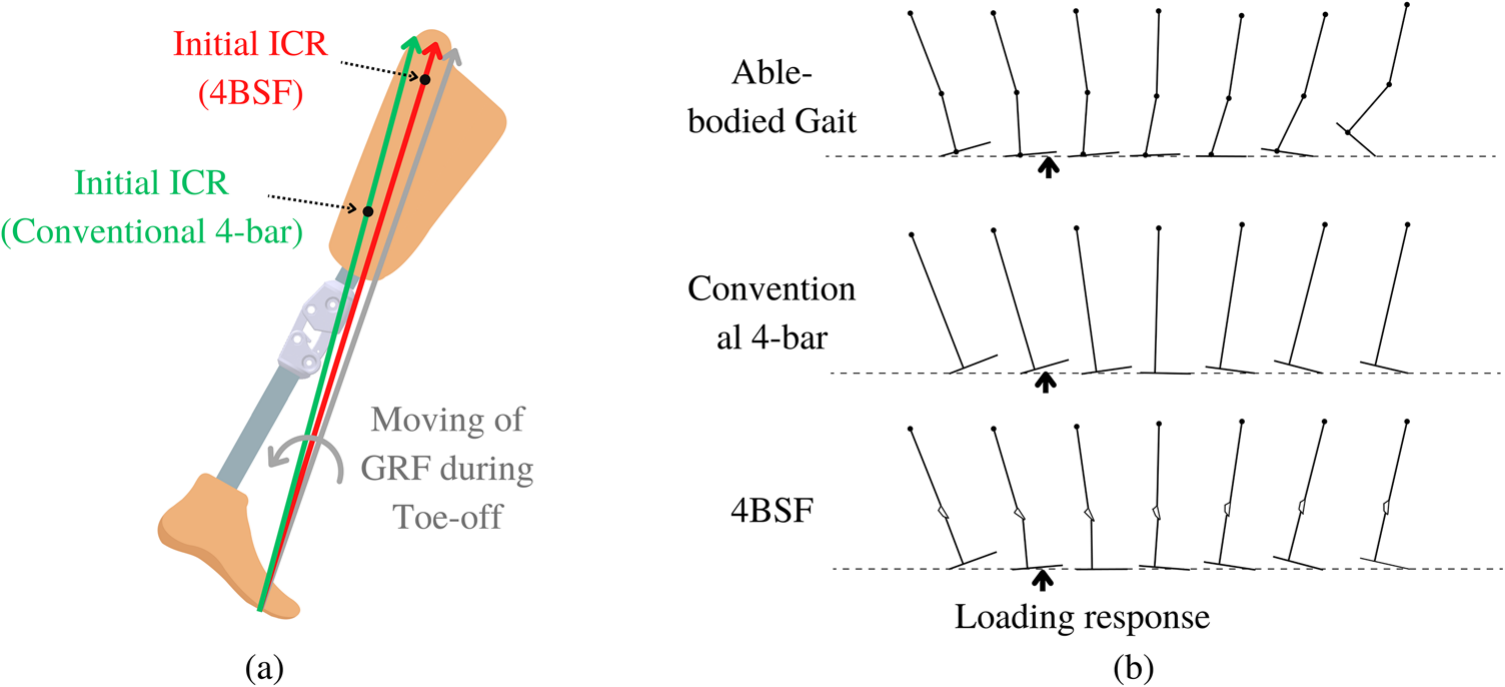
(**a**) The comparison of initial ICR between the 4BSF and the conventional 4-bar knee, (**b**) Comparison of gait analysis between able-bodied, conventional 4-bar, and 4BSF.

The transition from the stance phase to the swing phase of the conventional 4-bar was slower than the 4BSF because of the difference in the initial ICR position between each mechanism, as shown in Fig. 8a. At toe-off, the GRF initially acts in front of the ICR positions of both the 4BSF and conventional 4-bar mechanisms, as indicated by the grey line. Subsequently, the GRF passes the initial ICR of the 4BSF (as shown by the red line) before reaching the initial ICR of the conventional 4-bar (as shown by the green line). This enables a faster transition to the swing phase in the 4BSF as compared to the conventional 4-bar polycentric knee. Therefore, the knee flexion angle profile of the 4BSF was closer to the profile of able-bodied gait than the conventional 4-bar.

### Foot to ground angle

Figure 7b shows that the early and longer duration time of foot flat gave more stance phase stability. The foot of the able-bodied gait tended to enter the foot flat at 5% until 45% of the gait cycle. On the other hand, the foot of the conventional 4-bar tended to enter the foot flat at 30% to 41% of the gait cycle. In comparison, the foot of the 4BSF tended to enter the foot flat at 21% until 35% (high stiffness), 14% until 35% (medium stiffness), and 14% until 35% (low stiffness) of the gait cycle.

As analyzed in Fig. 8b, the gait cycle was simulated at equal hip angles with three conditions. The able-bodied has stance phase knee flexion and ankle joint. The conventional 4-bar has neither stance phase knee flexion nor ankle joint, and the 4BSF only has stance phase knee flexion. The results of the simulation were used to explain the experimental results^28^.

According to Fig. 8b, the foot of an able-bodied gait was the fastest to enter the flat foot because it had a knee joint for the stance-phase knee flexion and an ankle joint for plantar flexion. It contrasts with people with amputations in developing countries who use both conventional 4-bar knee and SACH foot without knee and ankle flexion angles. As a result, their feet entered the flat foot slower than able-bodied gait, resulting in lower stability during early stance due to a lack of a stable base of support^25–27^. Although the foot of the 4BSF entered the flat foot slower than the able-bodied did, it entered the flat foot faster than a conventional 4-bar polycentric knee (Ottobock 3R20) 9%, 16%, and 16% of the gait cycle for high, medium, and low stance-phase spring stiffness, respectively.

In summary, Table 2 presents an overview of the design specifications. The prototype 4BSF was in accordance with the design specification.

**Table 2:** Evaluation of the prosthetic knee design specification compared to the prototype.

	Design specification	Prototype	Achievement
Maximum stance-phase knee flexion angle	Over 10°	0°–15°	Good
Maximum swing-phase knee flexion angle	Over 60°	110°	Good
Weight	Less than 1230 g	1150 g	Good
Length	Less than 188 mm	161 mm	Good

## Conclusions

A novel 4-bar polycentric knee called 4BSF has been designed to perform stance-phase knee flexion. A prototype was manufactured for this study. Gait analysis of a single-subject pilot study was conducted to prove the conceptual design and walking performance. It found that the result of the ICR position and GRF vector of 4BSF are consistent with the conceptual design. The walking performance was compared between a 4BSF and a conventional 4-bar polycentric knee with a SACH foot. The 4BSF can perform a stance-phase knee flexion angle of 5°–10°, while a conventional 4-bar polycentric knee has no stance-phase knee flexion. Moreover, the amount of time to achieve the foot flat of the 4BSF was faster, and the foot flat duration time was twice as long as the conventional 4-bar polycentric knee.

## Limitations and future work

The limitation for improvement in this study is the inability to directly measure the ICR path of the 4-bar polycentric knee. Future research could involve increasing the number of participants and comparing the performance of the 4BSF to other polycentric knee designs. Additionally, collecting data during uneven ground, stair and slope walking and evaluating parameters such as energy consumption, symmetric gait, and subjective questionnaires could provide valuable insights.

## Data Availability

The datasets generated and analyzed during the current study are not publicly available due to ethical limitations and privacy but are available from the corresponding author (P.T.) on reasonable request.
